# Impact of the 8th edition of the AJCC TNM classification on gastric cancer prognosis—study of a western cohort

**DOI:** 10.3332/ecancer.2020.1124

**Published:** 2020-10-15

**Authors:** Mariana Peyroteo, Pedro Carvalho Martins, Rita Canotilho, Ana Margarida Correia, Catarina Baía, Alexandre Sousa, Donzília Brito, José Flávio Videira, Lúcio Lara Santos, Abreu de Sousa

**Affiliations:** 1 Surgical Oncology Department, Instituto Português de Oncologia do Porto Francisco Gentil, EPE, Porto, 4200-072, Portugal; 2 Experimental Pathology and Therapeutics Group, Instituto Português de Oncologia do Porto Francisco Gentil, EPE, Porto, 4200-072, Portugal; a https://orcid.org/0000-0002-0941-2533

**Keywords:** gastric cancer, staging, eighth edition TNM classification, AJCC

## Abstract

**Introduction:**

The 8th edition of the American Joint Committee on Cancer (AJCC) TNM classification for gastric cancer introduced changes, mainly in stage III, with the incorporation of the pN3 sub-classification in the final staging group. The goal was to compare the 7th and 8th editions to evaluate the discriminative capacity of the new edition.

**Methods:**

This study was a retrospective review of patients with gastric cancer treated with surgery in 2013 and 2014.

**Results:**

We analysed 310 patients, with a median age of 66 years and out of which 55.5% were male. The most commonly performed surgery was subtotal gastrectomy (*n* = 158; 51%), with a median of 30 lymph nodes removed. With a median follow-up of 39.5 months, the 1- and 3-year overall survival (OS) was 82% and 59%, respectively. In stage III (*n* = 115), there was stage migration in 40 cases (34.8%), with upstage in 11 cases and downstage in 29 cases. In this group, there was a statistically significant difference in OS between N3a and N3b patients (*p* = 0.002), as well as a statistically significant difference in OS between stages IIIA, IIIB and IIIC when the 8th edition was applied (*p* = 0.001), which was not verified with the 7th edition (*p* = 0.057). In multivariate analysis, both extracapsular extension and N classification from TNM were independent prognostic factors (*p* = 0.033 and *p* = 0.024, respectively).

**Conclusion:**

The 8th edition of the AJCC TNM classification allows for a better prognostic refinement, namely in the new stage III groups after the stratification of lymph node disease in N3a and N3b. Factors that evaluate the biological behaviour of the disease remain excluded from this edition, such as extracapsular extension, which had a prognostic impact in our series.

## Background

Gastric cancer is the fifth most common malignant tumour worldwide, as well as the third cause of cancer-related mortality [1–3]. Despite the evolution of the treatment strategy in recent decades, namely, with the association of perioperative chemotherapy, prognosis remains poor, especially in locally advanced disease [4–6].

The American Joint Committee on Cancer (AJCC) TNM staging system is the most frequently used staging method for malignant diseases. The 8th edition of the AJCC classification for gastric cancer introduced significant changes, mainly in stage III [7, 8]. Stage I remained unchanged, but in stage II there was a migration of T1N3bM0 patients from stage IIB to IIIB. Regarding stage III, although in the 7th edition the N3 group was divided between N3a (7–15 positive lymph nodes) and N3b (more than 15 positive lymph nodes), this subdivision was not taken into account in the final staging. In the 8th edition, this N3 group subdivision was incorporated in order to improve survival stratification in these patients. On the other hand, in contrast to other malignant tumours like breast cancer, other factors that could allow for a better prognostic refinement, namely related to the biological behaviour of the disease, remain excluded from this revision of the AJCC classification for gastric cancer [8].

The main goal of this study was to compare the 7th and 8th editions of the AJCC TNM classification for gastric cancer in order to determine the proportion of patients in which stage migration would occur and to determine if the greater prognostic refinement introduced by the new edition would be verified in our population. The secondary goal was to establish prognostic factors that could improve the discriminative power of this classification.

## Methods

The data from all patients with gastric adenocarcinoma consecutively treated with surgery at Instituto Português de Oncologia do Porto, EPE, between January 2013 and December 2014, were retrospectively reviewed. All adult patients (≥18 years old) with histologic confirmation of adenocarcinoma who submitted to surgery with curative intent were included. Exclusion criteria were stage IV at diagnosis, recurrent gastric cancer, other synchronous malignant tumours or oesophagogastric junction tumours of Siewert types I and II [9].

Data regarding demographic features, treatment, histology and follow-up were recorded. The type of lymph node dissection carried out was classified according to the Japanese Gastric Cancer Association classification [10].

Statistical analysis was carried out with version 24 of the SPSS® software, with a *p* < 0.05 considered as statistically significant. Continuous variables were presented as median and range. Categorical variables were analysed using Pearson *χ*^2^ test or Fisher’s exact test as appropriate. Tumours were staged according to the 7th and 8th editions of the AJCC TNM classification for gastric cancer [7, 8]. Kaplan–Meier’s survival curves were built and stratified according to both editions and compared using the log-rank test.

A univariate analysis was carried out in stage III patients to test the association between potential prognostic factors (independent variables) and the dependent variable, which was overall survival (OS) in this study. The analysed independent variables were age, gender, type of lymph node dissection, number of removed lymph nodes, lymphovascular invasion in the primary tumour, perineural invasion in the primary tumour, T and N classification of the TNM system and extracapsular extension in removed lymph nodes. The factors that achieved statistical significance in the univariate analysis were used to build the multivariate analysis model, through Cox regression, with hazard ratio (HR) and the respective 95% confidence intervals (95% CI) reported.

## Results

A total of 310 patients were included in the analysis, out of which 55.5% (*n* = 172) were male patients. The median age was 66 years (range: 24–89). The characteristics of the study group are described in Table 1.

The surgery performed was subtotal gastrectomy in 51% (*n* = 158) of cases, total gastrectomy in 47.1% (*n* = 146) and other types of surgery in 1.3% (*n* = 6), specifically gastrectomy totalisations (after previous antrectomy for peptic ulcer disease) and one superior polar gastrectomy (fundus and corpus resection for a proximal gastric tumour). The type of lymph node dissection was specified in 74.8% (*n* = 232) of cases, with D2 lymphadenectomy made in 36.2% (*n* = 84) of cases, D1+ in 60.8% (*n* = 141) and D1 in 3% (*n* = 7). Neoadjuvant chemotherapy was carried out in 6.5% (*n* = 20) of the patients and adjuvant chemotherapy in 31.7% (*n* = 98).

Regarding histological analysis, the median tumour size was 4.5 cm (range: 0.99–16.5), with lymphovascular invasion in 62.3% (*n* = 193) of tumours and perineural invasion in 46.1% (*n* = 143). Multifocality was found in 4.8% (*n* = 15) of cases and the most common histological subtype was intestinal (55.2%; *n* = 171). Concerning pathological staging, 29% (*n* = 90) of tumours were classified as pT1 (32 as pT1a and 58 as pT1b), 17.4% (*n* = 54) as pT2, 32.3% (*n* = 100) as pT3 and 21.3% (*n* = 66) as pT4 (59 as pT4a and 7 as pT4b).

A median of 30 lymph nodes (range: 5–95) was retrieved in the lymph node dissection product, with 52.6% (*n* = 163) of patients with positive lymphadenectomies, with a median of five positive lymph nodes (range: 0–65). Extracapsular extension was identified in 26% (*n* = 75) of cases. Regarding pathological staging, 47.4% (*n* = 147) of tumours were classified as pN0, 18.4% (*n* = 57) as pN1, 11% (*n* = 34) as pN2 and 23.2% (*n* = 72) as pN3 (49 as pN3a and 23 as pN3b).

**Figure 1.**
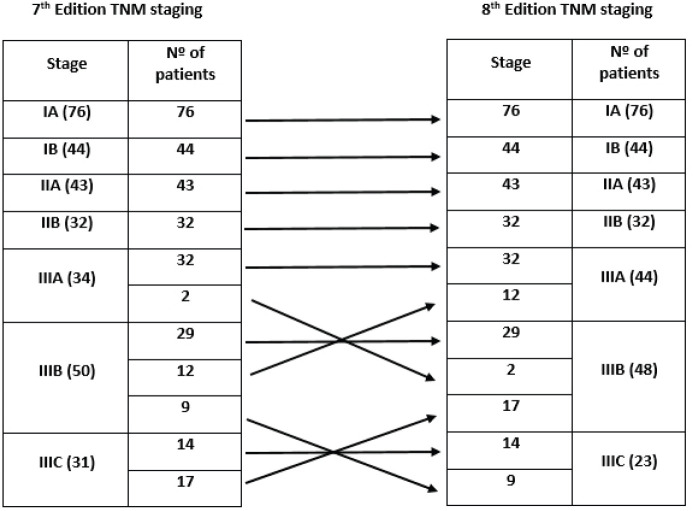
Distribution according to the 7th and 8th editions of the AJCC TNM classification (Nº: number).

The distribution of stage groups according to the 7th and 8th editions of the AJCC TNM classification is shown in Figure 1. The distribution in our cohort was as follows: stage I, 38.7% (*n* = 120) of cases; stage II, 24.2% (*n* = 75); and stage III, 37.1% (*n* = 115). Regarding stage migration, there were no changes between the two editions in stages I and II. Differences in staging from the 7th to 8th edition were verified in 40 patients (12.9%), all in stage III, with upstage in 11 cases (two from IIIA to IIIB and nine from IIIB to IIIC) and downstage in 29 cases (12 from IIIB to IIIA and 17 from IIIC to IIIB).

**Figure 2.**
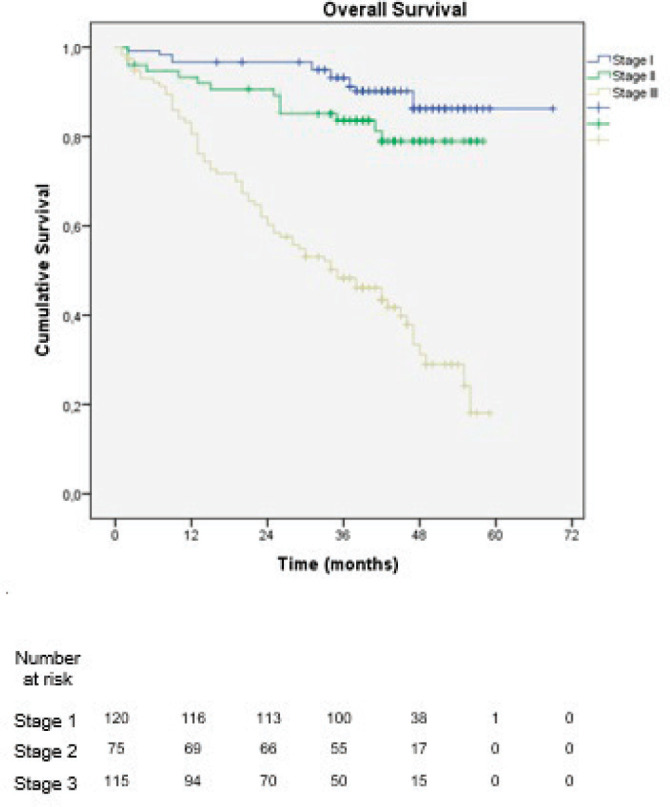
Overall survival stratified by staging according to the 8th edition of the AJCC TNM classification.

With a median follow-up of 39.5 months, 1- and 3-year OS was 82% and 59%, respectively. When stratified by stage groups, the 3-year OS was 87% in stage I, 79% in stage II and 21% in stage III, with a statistically significant difference in OS between stages (*p* < 0.001) (Figure 2).

**Figure 3.**
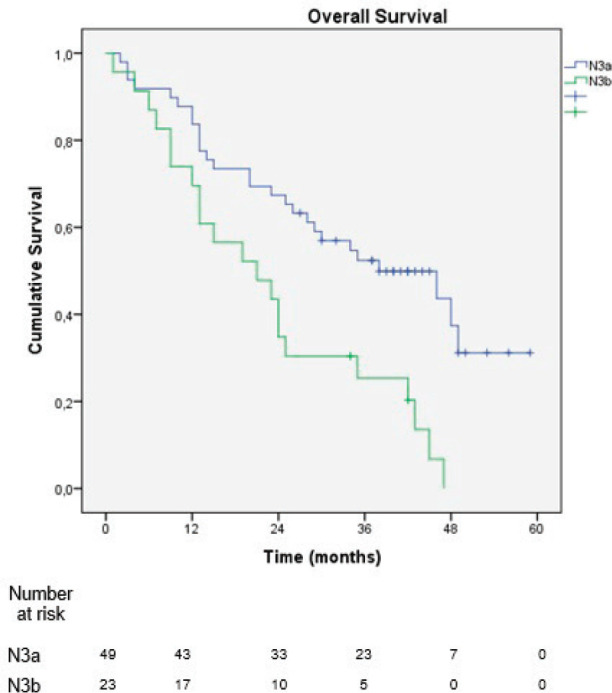
Overall survival stratified by the subdivisions N3a and N3b of the 8th edition of the AJCC TNM classification.

**Figure 4.**
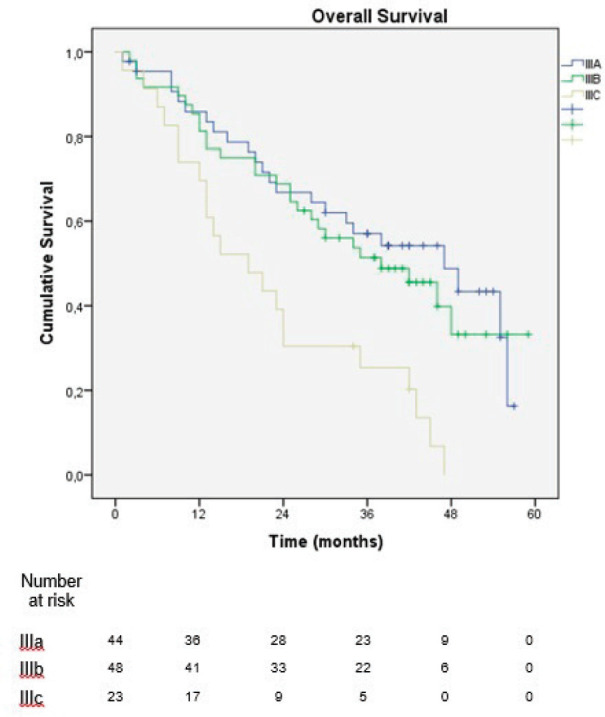
Overall survival of stage III patients according to the 8th edition of the AJCC TNM classification.

**Figure 5.**
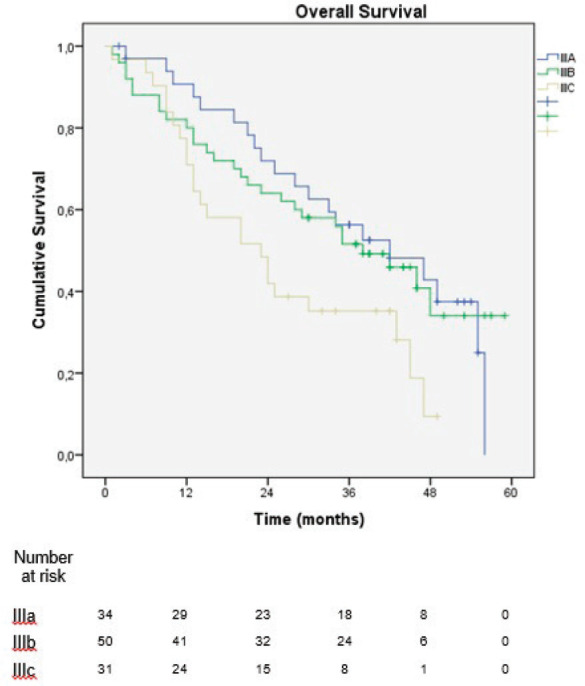
Overall survival of stage III patients according to the 7th edition of the AJCC TNM classification.

In the sub-analysis of stage III patients (*n* = 115), there was a statistically significant difference in OS between N3a and N3b patients (*p* = 0.001) (Figure 3). A statistically significant difference in OS between stages IIIA, IIIB and IIIC was found when the 8th edition was applied (*p* = 0.001), but not when the 7th edition was used (*p* = 0.057), (Figures 4 and 5). In the univariate analysis of potential prognostic factors in stage III patients (*n* = 115), extracapsular extension and the number of positive lymph nodes (N classification form TNM) had a statistically significant relationship with OS (*p* = 0.02 and *p* = 0.007, respectively). In multivariate analysis, both of these factors (extracapsular extension and N classification from TNM) were independent prognostic factors (*p* = 0.033 and *p* = 0.024, respectively), (Table 2).

## Discussion

The 8th edition of the AJCC TNM classification introduced important changes, mainly in stage III patients, with the incorporation of the N3a and N3b subdivision in the final staging [8]. In our series, stage III corresponded to a significant percentage of patients (37.1%), being the only stage where migration occurred. In fact, there was a statistically significant difference in OS between N3a and N3b patients, which probably explains the 40 cases (12.9%) of stage migration in stage III (upstage in 11 cases and downstage in 29 cases).

The new edition of the AJCC TNM classification for gastric cancer was largely based on the proposal by Sano *et al* [11], which was based on a mostly eastern population, raising the question of its applicability in western populations, which is similar to what happened in the transition from the 6th to 7th edition of the classification [12, 13]. However, the greater discriminative power of the 8th edition was verified in our cohort by the statistically significant difference in OS between stages IIIA, IIIB and IIIC, which was demonstrated when the 8th edition was applied (*p* = 0.001) but not when the 7th edition was used (*p* = 0.057). The present series is one of the few studies, together with Haejin In *et al* [14] and Graziosi *et al* [15], that evaluated western populations and came to similar conclusions.

The changes introduced by the new edition of the classification reinforce the importance of lymph node metastasis as a prognostic factor in gastric cancer and, in consequence, of the number of lymph nodes retrieved as an essential factor for accurate staging [16–20]. In fact, despite still not clearly establishing the minimum number of lymph nodes that should be retrieved in surgery, the inclusion of the subdivisions N3a and N3b in the final staging implies the resection of at least 15 lymph nodes in order for the N3b classification to be attributed. In our series, a median of 30 lymph nodes was retrieved, which follows the trend of this new edition regarding the better discriminative power associated to more extensive lymphadenectomies. This was also verified in the series by Lu *et al* [21], with the finding that the 8th edition of the classification had a slightly greater discriminative power when >30 lymph nodes were removed.

On the other hand, the new edition still excludes other prognostic factor that could contribute to a better prognostic refinement. Specifically, in our series, extracapsular extension was an independent prognostic factor. Extracapsular extension has been previously established as a poor prognostic factor both in early and advanced stages of gastric cancer [22, 23], with potential implications in adjuvant chemotherapy indications in early stages [24]. So, these findings suggest that this could be a factor to include in future revisions of the classification, since it is easily accessible information through histological observation alone. Besides, the inclusion of other factors related to the biological behaviour of the disease in the staging system could help select patients for adjuvant/perioperative chemotherapy, as well as define the adequate type of lymphadenectomy, since despite the changes introduced by the new edition, these do not have implications in defining the treatment strategy as yet.

One of the main limitations of this study is related to its retrospective nature, which explains why we did not have access to all the registries of the type of lymphadenectomy carried out. In patients where we could define the type of lymphadenectomy, D2 dissection was carried out in only 36.2% of cases and D1+ was carried out in the majority of patients (60.8%). This is related to the evidence available at the time of the study period, establishing that D2 dissection was not superior to D1+ in terms of OS [25]. In addition, the median of 30 lymph nodes resected attests to a good lymphadenectomy in our patients.

Finally, other limitations are related to the study being unicentric, with a follow-up time that can be insufficient for bigger conclusions and with the inclusion of few patients submitted to perioperative chemotherapy, which is related to the study period and may influence the survivals presented. On the other hand, it paints a better picture of the natural behaviour of this disease, with the possibility of serving as a benchmark for future studies.

## Conclusion

The new staging system introduced by the 8th edition of the AJCC TNM classification adds value and discriminative power in comparison to the previous edition in patients with gastric adenocarcinoma treated with surgery. This represents a significant improvement, especially in stage III patients, due to the better stratification of lymph node disease between N3a and N3b. However, these efforts did not go as far as what was presented for other malignancies in this edition, and it was demonstrated here that the inclusion of other factors that evaluate the biological behaviour of this disease, like extracapsular extension, could have further refined this tool.

## Conflicts of interest

The authors have no conflicts of interest to declare.

## Funding

There is no funding to declare.

## Authors’ contributions

MP and AS idealised the study concept, designed the methodology and wrote the article. PCM contributed to revising the article and statistical analysis. RC, AMC and CB contributed to writing the first draft of the article. DB, JFV, LLS and ADS reviewed the final draft of the article.

## Figures and Tables

**Table 1.** Descriptive characteristics of the study cohort (IQR: interquartile range; Nº: number).

Characteristics	Values
Age, years, median (IQR)	66 (57–74)
Gender, *n* (%) Female Male	138 (44.5%) 172 (55.5%)
Type of gastric surgery, *n* (%) Subtotal gastrectomy Total gastrectomy Other procedures	158 (51%) 146 (47.1%) 6 (1.9%)
Type of lymph node dissection (*n* = 232), *n* (%) D2 D1+ D1	84 (36.2%) 141 (60.8%) 7 (3%)
Neoadjuvant chemotherapy, *n* (%) No Yes	290 (93.5%) 20 (6.5%)
Adjuvant chemotherapy, *n* (%) No Yes	212 (68.3%) 98 (31.7%)
Size of the tumour, cm, median (IQR)	4.5 (3–7)
Lymphovascular invasion, *n* (%) No Yes	117 (37.8%) 193 (62.2%)
Perineural invasion, *n* (%) No Yes	167 (53.9%) 143 (46.1%)
Multifocality, *n* (%) No Yes	295 (95.2%) 15 (4.8%)
Histological subtype, *n* (%) Intestinal Mixed Signet ring cells Mucinous	171 (55.2%) 72 (23.2%) 64 (20.6%) 3 (1%)
pT, *n* (%) pT1a pT1b pT2 pT3 pT4a pT4b	32 (10.3%) 58 (18.7%) 54 (17.4%) 100 (32.2%) 59 (19%) 7 (2.3%)
Nº of retrieved lymph nodes, median (IQR)	30 (23–40)
Nº of positive lymph nodes, median (IQR)	5 (0–5)
pN, *n* (%) pN0 pN1 pN2 pN3a pN3b	147 (47.4%) 57 (18.4%) 34 (11%) 49 (15.8%) 23 (7.4%)

**Table 2.** Univariate and multivariate analysis of prognostic factors for OS (CI: confidence interval; HR: hazard ratio; Nº: number).

Prognostic factors for OS				
	Univariate analysis		Multivariate analysis	
	HR (95% CI)	*p*-value	HR (95% CI)	*p*-value
Age	1.01 (0.99–1.03)	*p* = 0.411		
Gender (male)	1.22 (0.75–1.97)	*p* = 0.423		
Lymphovascular invasion	1.54 (0.67–3.56)	*p* = 0.315		
Perineural invasion	1.30 (0.74–2.27)	*p* = 0.367		
Size of tumour (≥4.5 cm)	1.18 (0.69–2.04)	*p* = 0.549		
Type of lymph node dissection D1 D1+ D2	1 0.32 (0.10–1.07) 0.36 (0.11–1.21)	**** *p* = 0.179		
Nº of lymph nodes removed (≥30)	0.88 (0.55–1.42)	*p* = 0.598		
**Extracapsular extension**	1.96 (1.11–3.45)	***p* = 0.020**	1.84 (1.02–3.32)	***p* = 0.033**
T T2 T3 T4a T4b	1 1.04 (0.25–4.35) 1.70 (0.41–7.11) 1.08 (0.10–5.93)	**** *p* = 0.236		
**N** N0 N1 N2 N3a N3b	1 2.95 (0.38–23.28) 1.70 (0.22–13.33) 2.29 (0.31–17.09) 5.52 (0.72–42.13)	***p* = 0.007**	1 2.08 (0.25–17.02) 0.86 (0.10–7.22) 0.88 (0.10–7.48) 1.33 (0.13–13.37)	***p* = 0.024**
